# Impulsive UV-pump/X-ray probe study of vibrational dynamics in glycine

**DOI:** 10.1038/s41598-018-33607-4

**Published:** 2018-10-18

**Authors:** Riccardo Mincigrucci, Markus Kowalewski, Jérémy R. Rouxel, Filippo Bencivenga, Shaul Mukamel, Claudio Masciovecchio

**Affiliations:** 1 Elettra Sinctrotrone Trieste SCpA, Strada Statale 14 - km 163, 5, 34149 Basovizza, Trieste Italy; 2 Department of Chemistry, University of California, Irvine, California 92697-2025 USA

## Abstract

We report an ab-initio study of a pump-probe experiment on the amino-acid glycine. We consider an UV pump followed by an X-ray probe tuned to carbon K-edge and study the vibronic structure of the core transition. The simulated experiment is feasible using existing free electron laser or high harmonic generation sources and thanks to the localization of the core orbitals posseses chemical selectivity. The present theory applies to other experimental schemes, including the use of a THz probe, available with present soft X-ray free electron lasers and/or high harmonic generation sources.

## Introduction

**Figure 1:**
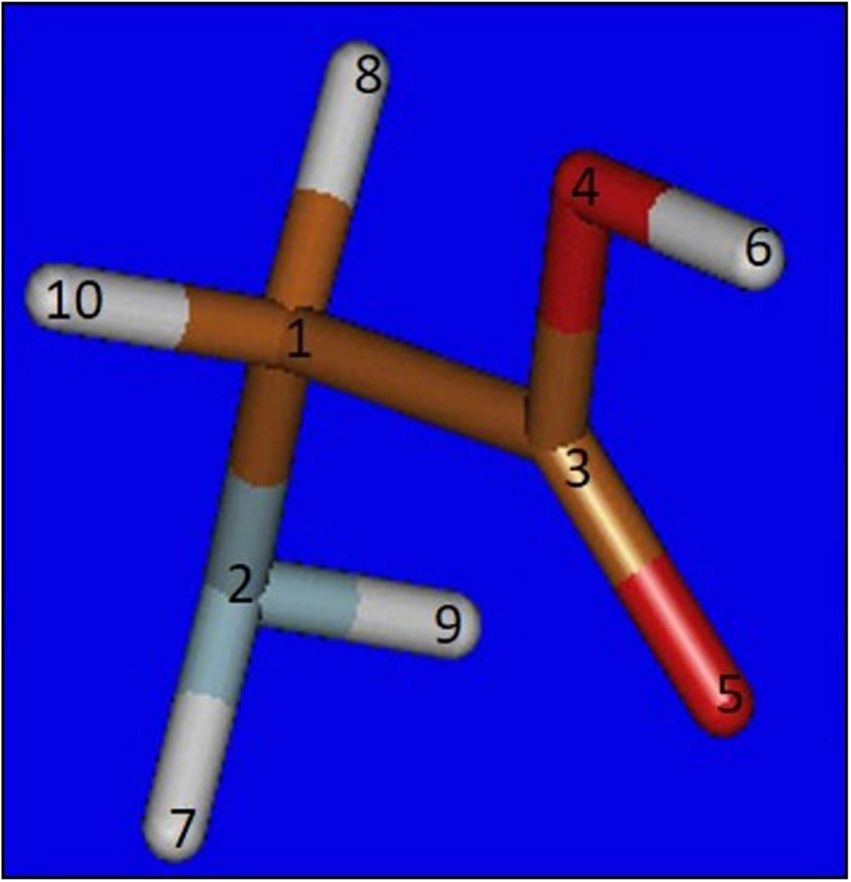
The glycine structure considered in the present work.

Spectroscopic techniques aim at revealing different and detailed matter properties. The simplest technique, linear absorption, can determine the energy levels of a system^1^ and by tuning the photon energy, different degrees of freedom can be investigated, e.g. vibrational and rotational transitions (in the infra-red spectral region)^2,3^ and electronic transitions^4,5^ (visible/X-ray spectral regions). The development of non-linear wave mixing has pushed forward the experimental capabilities, by adding selectivity and increasing the signal-to-noise ratio^6–8^. One of the simplest wave mixing approaches, is pump-probe (PP), which can impulsively trigger and monitor the ultrafast matter dynamics in real time. PP can be regarded as a special case of a third order process, where both the pump and the probe fields interact twice with the matter^9^. The promise to add chemical selectivity to the retrieved informations, thanks to the localization of the core transitions, has motivated the extension of the PP toward the X-ray spectral region. A number of experiments have been reported so far^10–15^ using high harmonic generation (HHG) and Free Electron Laser (FEL) sources. Despite tremendous efforts made to develop HHG sources culminating e.g. in ultra wide bandwidths^16^, they still provide a low flux, a limited wavelength tunability, and the polarization is not always precisely controlled^17–21^. In contrast, thanks to their higher brilliance, FEL sources have already demonstrated the capability to generate non-linear optical processes such as sum frequency generation^22^, second harmonic generation^23^, and transient grating^24^. In addition, broad wavelength tunability and multicolor options are available at existing FELs^25–27^, stimulating the theoretical description of multi-dimensional X-rays experiments. These theoretical approaches often assume pulses at the cutting edge, and even beyond, current technologies (sub-fs duration, heterodyne detection, multiple pulses with sub-fs delays and independent central wavelength, etc.) driving the development of new sources^27–29^. While the combination of theory and experimental capabilities is generally advisable to trigger any scientific advance, calculated signals and methodologies are increasingly needed to readily understand experiments and/or design measurements using the state-of-the-art technology. Here we apply, the non-linear response formalism^9^ to simulate the optical pump/X-ray probe signal of the simplest amino acid glycine (C_2_H_5_NO_2_), Fig. 1, in order to monitor impulsively excited vibrations: this experimental scheme is achievable within the current FEL technology. In our calculations glycine was considered to be in the gas phase. We have tuned the probe wavelength to the carbon K-edge and calculated the signal due to four vibrational modes.

## Results

**Figure 2:**
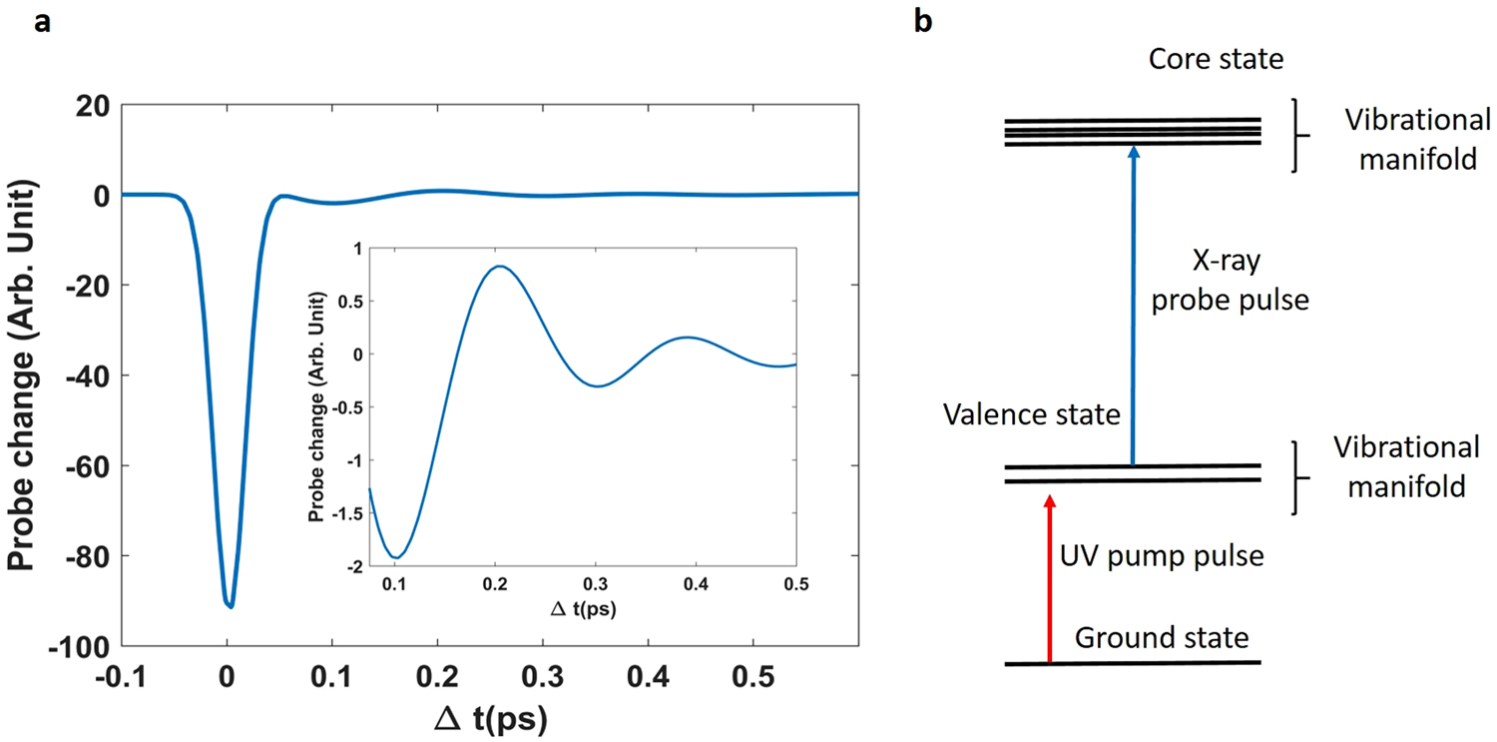
Modulation of the probe transmission by the four vibrations in our model (panel (a)). Inset is a zoom of the signal between 80 and 500 fs. Panel (b) gives the energy level scheme employed in this calculation.

**Figure 3:**
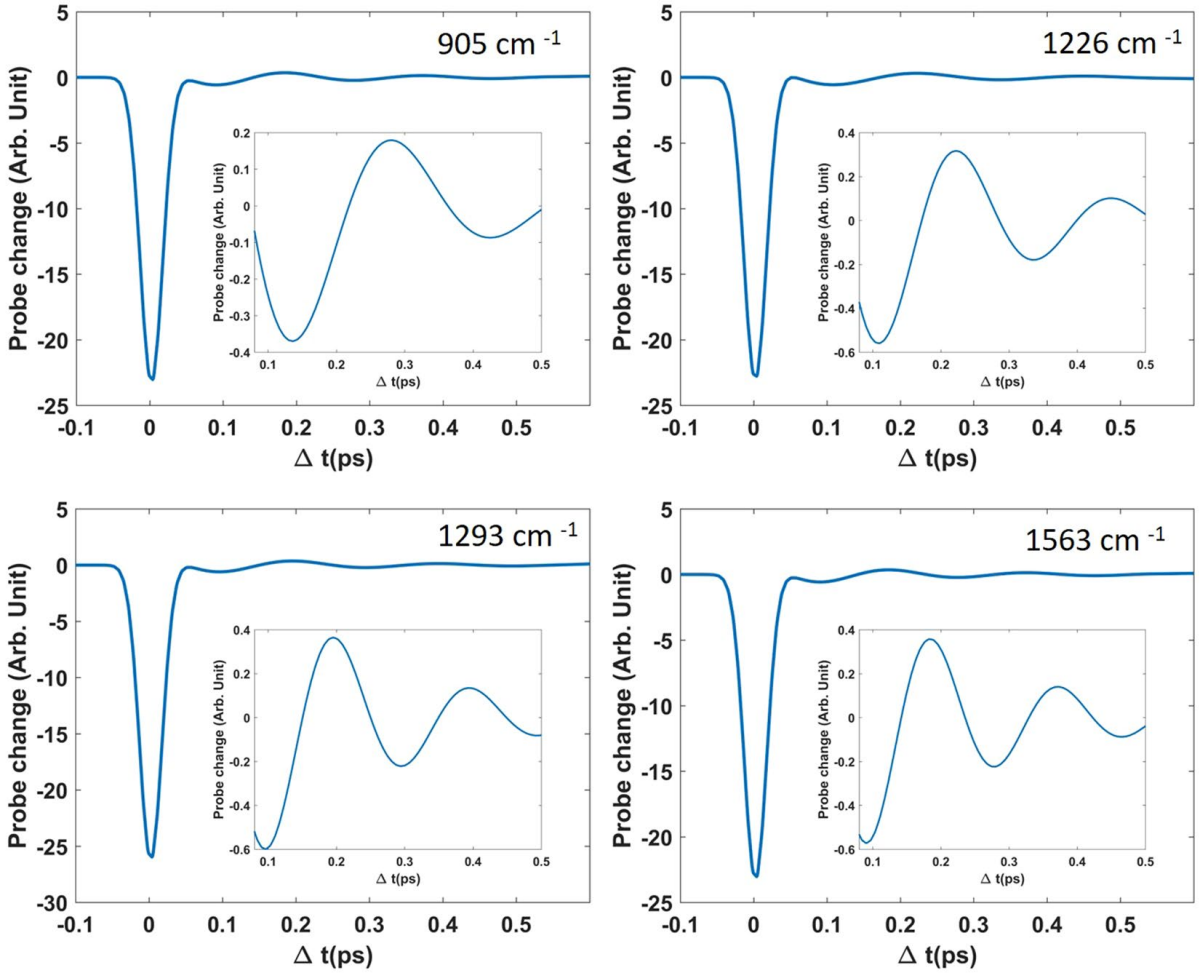
Pump-probe signals calculated for each of the selected vibrations. Insets are a zoom of the signal between 80 and 500 fs.

The signal shown in Fig. 2 was calculated by summing the contributions of the four vibrations shown in Fig. 3, considered as independent. All shown signals are characterized by a sharp initial drop, ascribable to the pulse cross correlation, followed by a modulation which amplitude is ~100 times smaller than the peak. The modulated part of the simulated signal (${\rm{\Delta }}t > 50$ fs) displayed in Fig. 2 shows that the proposed scheme is sensitive to vibrational coherences in the valence excited state.

In fact, closely examinating Eqs 3 and 4 is possible to see that coherences ($e\ne e^{\prime}$) are responsible for any oscillatory part of the signal, while populations ($e=e^{\prime}$) for intensity exponential decays. We found that, to probe the valence vibrational structure of the carbon K-edge transition, the probe must be tuned the energy difference between the first excited valence state and the core states of the carbon, i.e. ~286 eV. This is in agreement with a previous work where the highest sensitivity to vibrational dinamycs was observed in the pre-edge region^13^. In all calculations, we only considered the lowest energy core-hole state (see Table 2), however this approximation is well motivated by the fact that existing FEL sources^30^ can have sub-1 eV transform limited pulses that can be used to discriminate among the different states. At the same time, the FEL tunability enables to finely select such state and eventually change the probed atomic specie. Wide bandwidth pulses, as the one provided by HHG^16^, may be considered as well at the cost of longer computational times and a richer dynamic that has to be interpreted. Indeeed,the techniques considered here can also be applied to more complex molecules and/or molecular aggregates where the same atomic species can be subjected to different local environments and the core edges can be chemically shifted^31^ but still inside the pulse bandwidth.

## Discussion

**Figure 4:**
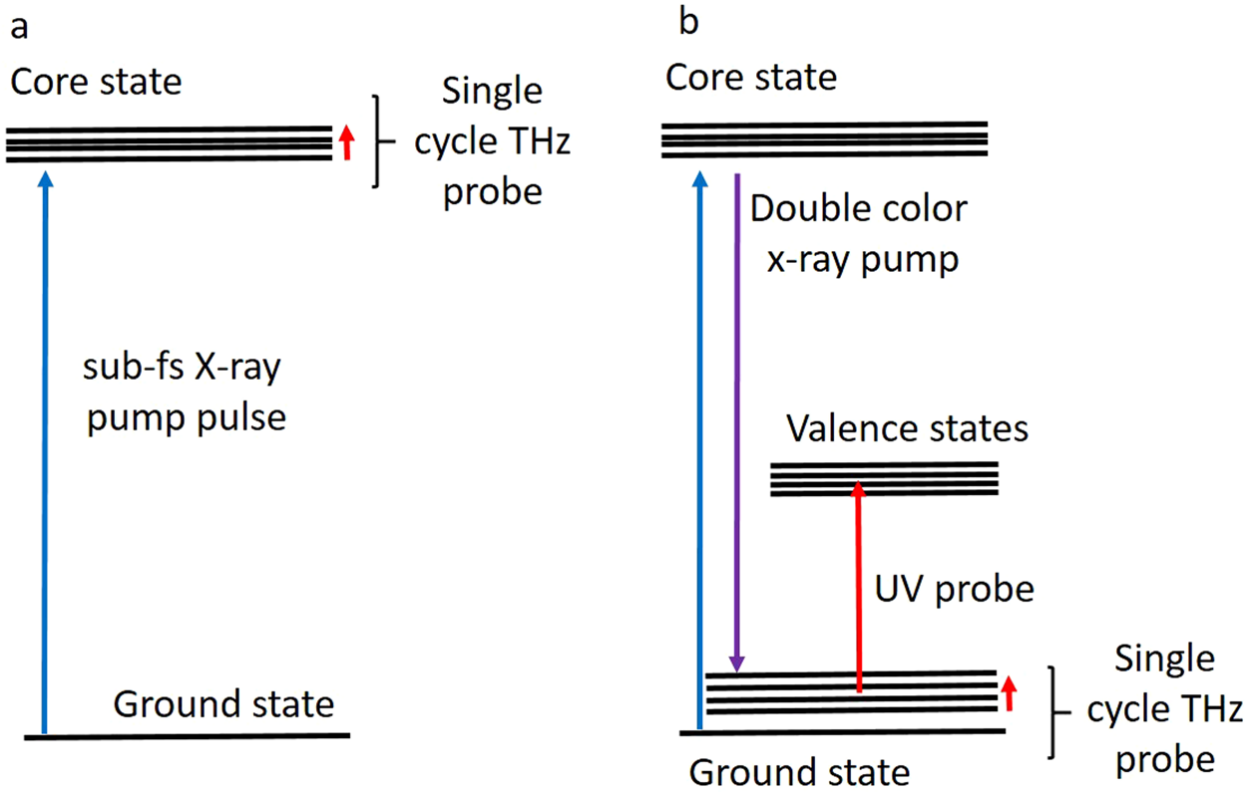
(**a**) An X-ray pump scheme beyond current technical possibilities and (**b**) the currently achievable pulse sequence.

The equations and steps presented in the current work can serve as a guideline to describe/calculate the expected signals for few more PP schemes, once that the relevant transition dipole moments have been computed. Indeed, in the presented scheme the active vibrations are those possessing a valence potential displaced with respect to the ground state, but reversing the pulse sequence (X-ray pump/UV probe) it could be possible to detect vibrational modes possessing a core displaced potential with respect to the ground state. However, due to the short lifetime of the carbon K-edge (3.3 fs), a more appropriate configuration is represented by sub-fs X-ray pulses combined with, e.g., a single cycle THz probe (Fig. 4a). In this case, a transient absorption of the THz probe can be detected. Both HHG and FEL facilities may envision to perform such kind of studies exploiting either the high repetition rate of the HHG sources or the high brilliance of the FEL ones, where THz emission are becoming available as a side-product of the FEL generation process^32^. Pushing further the imagination, it should be possible to design Raman-like processes, where an X-ray pulse creates ground state vibrational coherences for the modes presenting core displaced potentials (Fig. 4b). However, it is well known that removing an electron from a core orbital causes an instantaneous shift of the core energy^33^, but this difficulty can be overcome by exploiting time-coincident, tunable, double color emission: an option already available^25,30^. For such processes, the high brilliance of an FEL source would be essential since the pump role is hold by the X-ray pulses, while the obtained ground state vibrational coherences can subsequently be probed both with an UV pulse, i.e. using a valence transition, or with a THz one, as shown in Fig. 4b.

In conclusion, we have employed the nonlinear optical response formalism^9^ to calculate a resonant UV pump/X-ray probe signal which is sensitive to valence vibrational coherences. A specific application was made to the amino-acid glycine, which vibrational dynamics, especially in the dimer/trimer, form is under active investigation^34–37^ and, furthermore, thanks to its small size can be treated with high level quantum chemistry methods. The latter point is particularly relevant since it enables glycine to be used as a benchmark to compare experiments and theory. In fact, it has been proposed that photo-dissociation due to conical intersections may take place after UV excitation^38^, and an experiment as the one depicted here can certainly help to clarify this aspect.

## Methods

### Monitoring vibrational dynamics by the pump-probe technique

**Figure 5:**
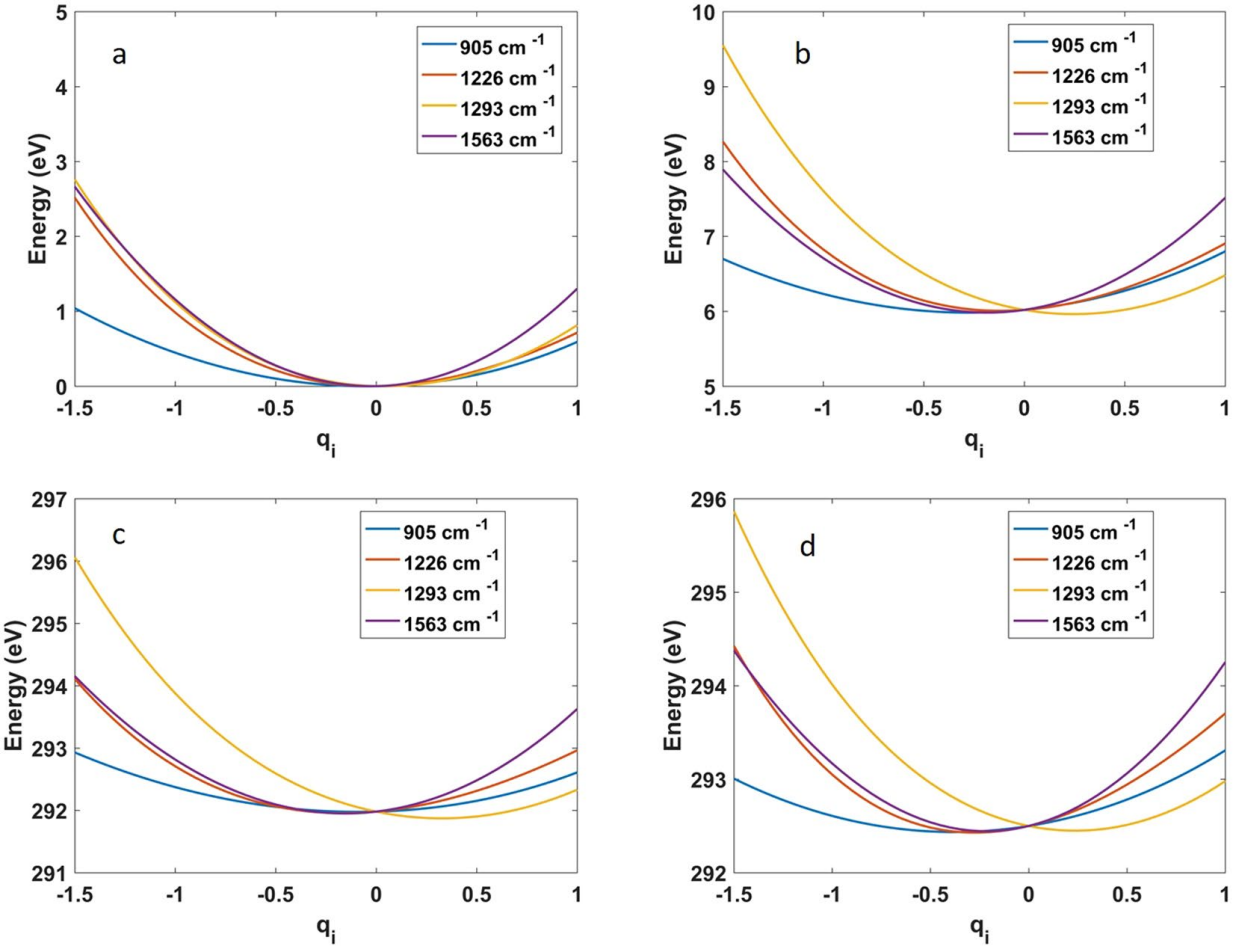
Electronic potentials calculated in the molecular ground state (**a**), first valence excited state (**b**), C_COOH_ (**c**) and ${{\rm{C}}}_{{{\rm{CNH}}}_{2}}$ (**d**) core states, for the four vibrations.

**Figure 6:**
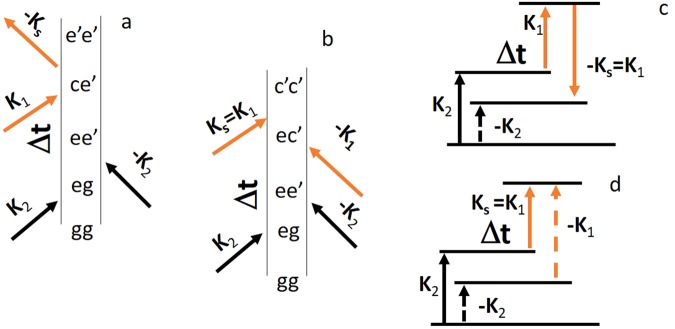
Ladder diagrams (**a**,**b**) and corresponding level schemes (**c**,**d**) for the PP signal: stimulated Raman (**a**,**c**) and stimulated absorption (**b**,**d**). Dashed/full lines in diagrams (**c**,**d**) represent an interaction with the bra/ket^51^. The complex conjugate diagrams (not shown) also contribute to the signal.

To determine the relevant vibrational manifold of glycine, we have calculated the valence electronic potential surfaces and transition dipoles (***μ***) at CASSCF(6/4)/6-31G* level of theory. Calculation of excited states potentials energy surfaces with CASSCF/6-31G* is wide spread practice and shows a good agreement with experiments^39,40^. Moreover, the electronic structure in our case is rather simple and does neither involve dissociative features, non-adiabatic couplings, or electron transfer features. Four vibrational modes (905, 1226, 1293, and 1563 cm^−1^) were selected from a normal mode analysis, based on their large displacement in the valence excited state. The selected modes include NH_2_ out-of-plane bending motion (905 cm^−1^), CO and CN stretch (1226 cm^−1^) CO and CC stretch (1263 cm^−1^), and CH_2_ out-of-plane bending motion (1563 cm^−1^)^41^. The anharmonic potentials of the ground *g*, valence excited *e*, and core states *c* of the modes are depicted in Fig. 5. The anharmonic vibrational eigenfunctions of each mode were calculated (single active mode approximation, see computational methods for further details) and the wave packet dynamics is described by expanding in these eigenstates. The PP signal was calculated using the non-linear response formalism^9^ by a sum over the eigenstates considering the relevant diagrams shown in Fig. 6b, together with (Fig. 6c,d) the corresponding transition level scheme. The first step of the process is photon absorption given by two interactions with the pump field (labelled **k**_2_ and −**k**_2_ in Fig. 6), which excites the molecule from *g*. The system is driven into a vibrational coherence (e ≠ e′) or population (e = e′) of the valence state. Finally after a time delay ${\rm{\Delta }}{\rm{t}}$, the coherences and populations are probed by the second pulse that induces a stimulated Raman scattering (diagram a–c, Fig. 6) or absorption (diagram b–d, Fig. 6).

The PP signal is defined as the total energy loss of the transmitted probe integrated over time^9^:

1 $${S}_{PP}^{n}({\rm{\Delta }}{\rm{t}})=\int \,{\rm{d}}t\frac{\langle d{H}_{{\rm{i}}{\rm{n}}{\rm{t}}}(t)\rangle }{dt}=-\,2{\omega }_{1}\text{Im}\,\int \,{\rm{d}}t{{\bf{P}}}^{(3)}(t,{\rm{\Delta }}{\rm{t}})\cdot {\bf{E}}(t,{\rm{\Delta }}{\rm{t}}),$$

where ${H}_{{\rm{int}}}(t)=-\,{\bf{P}}\cdot {\bf{E}}$ is the dipole coupling, ${{\bf{P}}}^{(3)}$ is the third order polarization, ${\omega }_{1}$ is the probe central frequency, ${\rm{\Delta }}{\rm{t}}$ is the pump-probe delay and n denotes the vibrational mode. Expanding the polarization perturbatively in the incoming fields, the signal, calculated separately for each vibrational mode, is given by

2 $${S}_{PP}^{n}({\rm{\Delta }}{\rm{t}})=2{\omega }_{1}\text{Im}\sum _{ee{\rm{^{\prime} }}cc{\rm{^{\prime} }}}\,{\int }_{-{\rm{\infty }}}^{{\rm{\infty }}}\,{\rm{d}}t\,{\int }_{0}^{{\rm{\infty }}}\,{\rm{d}}{t}_{1}{\rm{d}}{t}_{2}{\rm{d}}{t}_{3}({R}_{a}({t}_{3},{t}_{2},{t}_{1})+{R}_{b}({t}_{3},{t}_{2},{t}_{1}))G(t,{t}_{1},{t}_{2},{t}_{3};{\rm{\Delta }}{\rm{t}}),$$

where:

3 $${R}_{a}({t}_{3},{t}_{2},{t}_{1})=-\,\frac{i}{{\hslash }^{3}}{\langle {{\boldsymbol{\mu }}}_{e^{\prime} c}{{\boldsymbol{\mu }}}_{ce}{{\boldsymbol{\mu }}}_{e^{\prime} g}{{\boldsymbol{\mu }}}_{eg}\rangle }_{xxxx}{e}^{-i{\omega }_{1}{t}_{3}+i{\omega }_{2}{t}_{1}}{e}^{-i{\omega }_{ce^{\prime} }{t}_{3}-{\gamma }_{ce^{\prime} }{t}_{3}}{e}^{-i{\omega }_{ee^{\prime} }{t}_{2}-{\gamma }_{ee^{\prime} }{t}_{2}}{e}^{-i{\omega }_{eg}{t}_{1}-{\gamma }_{eg}{t}_{1}}$$

and

4 $${R}_{b}({t}_{3},{t}_{2},{t}_{1})=\frac{i}{{\hslash }^{3}}{\langle {{\boldsymbol{\mu }}}_{ce^{\prime} }{{\boldsymbol{\mu }}}_{ce}{{\boldsymbol{\mu }}}_{e^{\prime} g}{{\boldsymbol{\mu }}}_{eg}\rangle }_{xxxx}{e}^{i{\omega }_{1}{t}_{3}+i{\omega }_{2}{t}_{1}}{e}^{-i{\omega }_{e^{\prime} c}{t}_{3}-{\gamma }_{e^{\prime} c}{t}_{3}}{e}^{-i{\omega }_{e^{\prime} e}{t}_{2}-{\gamma }_{e^{\prime} e}{t}_{2}}{e}^{-i{\omega }_{ge}{t}_{1}-{\gamma }_{ge}{t}_{1}}$$

are the fourth rank response functions tensors containing the oscillatory part of the field. In Equations 3 and 4, ***μ*** are the transition dipole moments between the states, ${\omega }_{2}$ is the UV frequency, ${\omega }_{ij}$ are the matter frequencies, ${\gamma }_{ij}$ are the relative dephasing times and $\langle \ldots \rangle$ stands for the rotational averaging. Instead, *G* is the product of the field temporal envelopes:

5 $$G(t,{t}_{1},{t}_{2},{t}_{3};{\rm{\Delta }}{\rm{t}})={A}_{1}^{2}{A}_{2}^{2}{e}^{-\frac{{(t-{t}_{1}-{t}_{2}-{t}_{3}+{\rm{\Delta }}{\rm{t}})}^{2}}{2{\sigma }_{1}^{2}}}{e}^{\frac{-{(t-{t}_{2}-{t}_{3}+{\rm{\Delta }}T)}^{2}}{2{\sigma }_{1}^{2}}}{e}^{-\frac{{(t-{t}_{3})}^{2}}{2{\sigma }_{2}^{2}}}{e}^{-\frac{{t}^{2}}{2{\sigma }_{2}^{2}}}$$

where A_*j*_ are the electric field amplitudes and *σ*_*j*_ are the standard deviations. Generally speaking, the transition dipoles moments are cartesian vectors referred to a given molecular orientation. However, molecules in solutions or in powder form are randomly oriented and rotational averaging has to be performed on the *R*_*a*,*b*_.

Since both pulses were considered as linearly polarized along the *x* direction in the laboratory frame, the signal for each vibrational mode is given by the *xxxx* component of the rotationally averaged tensor:

6 $${\langle {\boldsymbol{\mu }}{\boldsymbol{\mu }}{\boldsymbol{\mu }}{\boldsymbol{\mu }}\rangle }_{xxxx}=\sum _{ijkl}\,\frac{1}{15}({\delta }_{ij}{\delta }_{kl}+{\delta }_{ik}{\delta }_{jl}+{\delta }_{il}{\delta }_{jk}){{\boldsymbol{\mu }}}_{i}{{\boldsymbol{\mu }}}_{j}{{\boldsymbol{\mu }}}_{k}{{\boldsymbol{\mu }}}_{l}.$$

Ultimately, the expected experimental signal is defined as:

7 $${S}_{PP}({\rm{\Delta }}{\rm{t}})=\sum _{n=1}^{4}\,{S}_{PP}^{n}({\rm{\Delta }}{\rm{t}}).$$

The signal intensity is expected to scale linearly with the pump pulse intensity and in principle high intensities are advisable to obtain a better experimental contrast. However, a thermodynamic estimation based on the formation enthalpy (~528 KJ/mol) permits to define the maximum tolerable pump flux to be ~1 photon per molecule. Selecting a pump resonant with the first valence transition defines the probe photon energy, necessary to obtain the resonant transition with the carbon atom, while the momentum conservation (phase matching) requires that the signal is emitted in the direction of the probe pulse. For each of the excited electronic states, we included in the simulation two vibrational eigenstates since the calculated Franck-Condon (FC)^42–44^ factors are negligible for higher states. The carbon K-edge core hole state lifetime (3.3 fs^45^) was included in the calculation as a broadening *γ*. The computed signal, Eq. 7, is shown in Fig. 2, panel a. In our calculations we assumed as experimental parameters a pump pulse of 6.1 eV 40 fs full width half maximum (FWHM) and a probe pulse of 286 eV, 40 fs FWHM corresponding to a bandwidth of 30 meV. A wider probe bandwidth may be accounted for by summing over a more extended set of states. However, in the present case this is not necessary since FC factors restricts the number of the accessible ones. The central frequency of the pump pulse is tuned to the sum of the pure electronic transition and the vibrational manifold. The employed photon energy, obtained by the ab-initio calculations, matches quite well the experimentally measured absorption peak for glycine^46^. The bandwidths of both pulses have been selected to cover a limited number of vibrational levels, in order to better illustrate the potential of this approach, and to meet existing experimental capabilities.

### Computational protocol

The Molden package^47^ was used to design the molecule and the geometry was subsequently optimized using MOLPRO^48^. Geometry optimization was obtained at the HF/6-31G* level of theory. The parameters, in good agreement with the experimental values^49^, obtained for the ground state are listed in Table 1.

**Table 1:** List of distances and angles obtained by the geometry optimization of glycine ground state.

Atoms	Distances (Å)	Atoms	Angles[°]
1–2	1.44	1-2-7	110.40
1–3	1.51	1-2-9	110.40
1–8	1.08	1-3-4	111.81
1–10	1.085	1-3-5	125.38
2–7	1.00	2-1-3	115.03
2–9	1.00	2-1-8	109.99
3–4	1.33	2-1-10	109.99
3–5	1.19	3-1-8	107.70
4–6	0.95	3-1-10	107.71
		3-4-6	108.38
		4-3-5	122.81
		7-2-9	106.21
		8-1-10	106.01

The valence electronic energy levels and transition dipoles have been calculated at the CASSCF(6/5)/6-31G* level of theory, where the active space has been selected in order to include the *π* and π* oxygen orbitals of the CO bond and lone pairs of the CO oxygen and the NH_2_ group. The core excited states of carbon atoms have been treated separately. To obtain the corresponding energy levels and transition dipole moments, the 1s orbital of each carbon atom is restricted to single occupancy (and frozen) to guarantee the convergence to a core-hole state. The new active space has been subsequently treated at the RASSCF(5/3) level of theory and the resulting energy levels are reported in Table 2.

**Table 2:** Energy levels calculated for the glycine isomer.

State	Energy (eV)
Valence (GND)	0
Valence	6,02
Core	292,6 [292,0]
Core	299,0 [295,3]

Values between square brackets refer to potentials relative to C in position 1.

The nuclear dynamics is calculated in the eigenbasis of the vibrational modes assuming a separable normal mode Hamiltonian:

8 $${\hat{H}}_{r}=\sum _{i=1}^{N}-\frac{1}{2{m}_{i}}\frac{{\partial }^{2}}{\partial {q}_{i}}+{\hat{V}}_{r,i}({q}_{i}),$$

where *m*_*i*_ is reduced mass and ${\hat{V}}_{r,i}$ the nuclear potential of the *i*^*th*^ normal mode in the *r*^*th*^ electronic state. The potentials ${\hat{V}}_{r,i}$ are obtained by displacing the equilibrium geometry **X**_*eq*_ by the cartesian displacement vector ${\boldsymbol{\Delta }}$***x***_*i*_ of the respective normal mode

9 $${{\bf{X}}}_{i}({q}_{i})={{\bf{X}}}_{eq}+{q}_{i}{\boldsymbol{\Delta }}{{\boldsymbol{x}}}_{i}$$

and calculate the single point energies in MOLPRO i.e. as the expectation values of the electronic states in the Born-Oppenheimer approximation:

10 $${V}_{r}({q}_{i})=\langle {\varphi }_{r}({q}_{i})|{\hat{H}}_{el}({{\bf{X}}}_{i}({q}_{i}))|{\varphi }_{r}({q}_{i})\rangle ;$$

This potential calculation was repeated for the four selected modes and the results are listed in Table 3. Ground state, first valence state and carbons potentials, as a function of the molecular coordinate *q* are shown in Fig. 5. The eigenfunctions ${\psi }_{r,i,m}({q}_{i})$ for each of the four modes have been calculated using the imaginary time propagation method on a numerical grid^50^ of the respective potential *V*_*r*_. In the calculation of the signal we only consider excitations in a single vibrational mode and assume that the other three modes are in the vibrational ground state:

11 $${{\rm{\Psi }}}_{r,i}(q)=\prod _{j\ne i}\,{\psi }_{r,0,j}({q}_{j})\,\sum _{m=0}^{M}\,{c}_{r,m}{\psi }_{r,m,i}({q}_{i})$$

with eigenvalues:

12 $${\omega }_{r,m,i}={E}_{r,m,i}+\sum _{j\ne i}^{N}\,{E}_{r,0,j}.$$

**Table 3:** Equilibrium positions and deformations for the the four selected vibrational modes.

Atom	X_*eq*_ (Å)	Δ*x*_905_ (Å)	Δ*x*_1226_ (Å)	Δ*x*_1293_ (Å)	Δ*x*_1563_ (Å)
1 (C)	−0.46, 0.16, −1.55	−0.01, −0.04, 0.15	0.17, 0.04, −0.03	0.04, 0.01, −0.01	−0.12, 0.01, −0.09
2 (N)	−1.88, −0.04, −1.64	0.06, 0.00, 0.04	−0.13, −0.03, 0.04	−0.03, −0.01, 0.02	0.00, −0.00, 0.02
3 (C)	0.15, −0.13, −0.20	−0.00, 0.03, −0.08	0.04, −0.00, 0.02	−0.12, −0.00, −0.06	0.13, −0.00, 0.07
4 (O)	1.46, 0.07, −0.19	−0.07, −0.01, 0.00	−0.06, −0.01, 0.00	0.12, 0.03, −0.04	−0.03, 0.00, −0.02
5 (O)	−0.45, −0.51, 0.76	0.06, 0.03, −0.08	0.01, 0.01, −0.011	−0.02, −0.02, 0.05	−0.02, 0.00, −0.01
6 (H)	1.79, −0.13, 0.68	−0.29, −0.07, 0.08	0.13, 0.04, −0.06	−0.47, −0.12, 0.17	−0.22, −0.05, 0.04
7 (H)	−2.35, 0.54, −0.97	−0.17, 0.07, −0.21	−0.25, 0.03, −0.11	−0.06, 0.02, −0.02	0.04, 0.07, −0.03
8 (H)	0.05, −0.46, −2.29	0.02, −0.3, 0.15	0.20, 0.04, −0.01	0.21, −0.01, 0.12	0.36, 0.00, 0.24
9 (H)	−2.11, −0.99, −1.41	−0.15, 0.00, −0.23	−0.24, −0.04, −0.13	−0.05, −0.02, −0.04	0.05, −0.03, −0.06
10 (H)	−0.21, 1.19, −1.81	0.02, −0.05, 0.14	0.20, 0.04, −0.01	0.21, 0.00, 0.13	0.37, −0.02, 0.23

The obtained eigenfunctions have been subsequently used to determine the FC factors that weight the electronic transition dipoles between the electronic states. The FC factors are given by

13 $${P}_{i,rs,mn}=\int \,{\rm{d}}q{\psi }_{r,i,m}^{\ast }(q){\psi }_{s,j,n}(q).$$

In Eq. 13, $\psi \,{(q)}_{r,i,m}$ is the *m*^*th*^ vibrational wave function of the *i*^*th*^ normal mode, calculated in the *r*^*th*^ electronic state. The transition dipole matrix elements are approximated by weighting the electronic transition dipole moment at the equilibrium geometry with the FC factors:

14 $${\mu }_{i,rs,mn}={\mu }_{rs}(q={q}_{eq}){P}_{i,rs,mn}.$$

No approximations or analytical integrations have been performed on the signal expression (Eq. 2). For each interaction pathways, i.e. sequence of *μ*’s and matter frequencies, the signal has been calculated for each ${\rm{\Delta }}$*t* value with Eq. 2, on the four dimensional space represented by the *t*, *t*_1_, *t*_2_, *t*_3_ tuple and summed. The following time intervals have been considered: $t\in [\,-\,50,50]$ fs, ${t}_{1},{t}_{2},{t}_{3}\in [0,500]$ fs.
